# The Usefulness of Dual-Layer Spectral Computed Tomography for Myelography: A Case Report and Review of the Literature

**DOI:** 10.1155/2018/1468929

**Published:** 2018-03-04

**Authors:** Yuji Iyama, Takeshi Nakaura, Ayumi Iyama, Kazuhiro Katahira, Yasuyuki Yamashita

**Affiliations:** ^1^Department of Diagnostic Radiology, Kumamoto Chuo Hospital, Kumamoto, Japan; ^2^Department of Diagnostic Radiology, Graduate School of Medical Sciences, Kumamoto University, Kumamoto, Japan; ^3^Department of Diagnostic Radiology, Japanese Red Cross Kumamoto Hospital, Kumamoto, Japan

## Abstract

We describe a case of lumbar stenosis in which retrospective spectral analysis using dual-layer spectral detector computed tomography (CT) had the ability to expand the evaluable region in the spinal canal. Spinal canal stenosis is a common condition whose symptoms (such as lower back and leg pain with walking) deteriorate the quality of life. Generally, magnetic resonance imaging (MRI) and CT myelography are performed to diagnose canal stenosis. Dual-layer spectral detector CT can yield virtual monochromatic imaging and retrospective on-demand spectral analysis without a prescan setting. Spectral analysis could expand the evaluable region in the spinal canal for increasing the contrast enhancement in the canal.

## 1. Introduction

Lumbar canal stenosis leads to compression of the thecal sac and may also involve the nerve roots because of narrowing of the intervertebral foramina. Computed tomography (CT) enhanced by myelographic contrast and magnetic resonance imaging (MRI) are well-known diagnostic tools for lumbar canal stenosis [1, 2].

In recent years, MRI has become the “gold standard” in the diagnosis of lumbar spinal stenosis because of its potential in visualizing radiolucent soft tissues and the absence of radiation exposure. However, MRI may not be useful for the evaluation of postoperative lumbar canal stenosis due to susceptibility artifact. In addition, MRI may be contraindicated in patients with pacemakers [3]. CT myelography is useful in these instances.

Recently, dual-energy CT has become clinically available [4, 5]. By means of the high- and low-energy X-ray spectra, dual-energy CT acquisition facilitates a greater degree of material characterization than the conventional single-energy acquisition. A previous report suggested the usefulness of dual-energy CT to improve image quality when assessing bone marrow edema because of the synthesizing virtual monochromatic images [6]. Virtual monochromatic images are particularly useful for evaluation of the differences in contrast enhancement between the spine and spinal canal. In CT myelography, the spinal cord is evaluated using an intrathecal contrast agent. However, the image quality in cervical and upper thoracic CT myelography is suboptimal in some cases [7]. Previous report suggested the usefulness of low-keV virtual monochromatic images for increasing the contrast enhancement for vascular and hepatic parenchyma [8]; therefore, dual-energy CT might increase the contrast enhancement in the canal. A previous report suggested the usefulness of dual-energy CT for the reduction of artifact and radiation dose [9] in CT myelography. However, it did not evaluate the utility of dual-energy CT in increasing the contrast enhancement of the spinal canal in CT myelography.

Recently, the first commercially available dual-layer spectral detector CT (IQon Spectral CT; Philips Healthcare, Best, Netherlands) has been introduced for clinical use. The scanner has a single X-ray source and two layers of detectors. Two layers collected the low-energy data and the high-energy data. This scanner enables us to acquire the low- and high-energy data simultaneously.

Here, we describe a case of lumbar canal stenosis in which retrospective on-demand spectral analysis using dual-layer spectral detector CT allowed a better evaluation of the thoracic and lumbar canal compared with conventional CT.

## 2. Case Report

A 73-year-old Asian male complained of bilateral buttock pain radiating into his thighs and calves. He could not walk for more than 10 min or 2-3 blocks due to pain. Initial patient consultation was made by the orthopedic department in our hospital. Laboratory data were unremarkable.

On physical examination, he was bilaterally positive for Lasègue's sign. MRI in our hospital showed multiple compression fractures (T10, T12, and L1) and spinal stenosis (L1-L2). The patient underwent CT myelography for a preoperative evaluation. The CT myelogram was performed following lumbar puncture at the L2-L3 level under fluoroscopy in the prone position and injection of 15 mL Omnipaque® 300 (iohexol) contrast. CT myelography was performed using a dual-layer spectral detector CT with a routine scan protocol. The scanning was started 10 min after contrast material injection. The scan parameters were as follows: detector configuration, 64 × 0.625 mm; gantry rotation time, 0.75 s; helical pitch (beam pitch), 0.578; tube voltage, 120 kVp; tube current time product, 162 mAs (effective mAs) with automodulation; and volume CT dose index, 13.9 mGy. This CT scan led to the diagnosis of lumbar canal stenosis.

**Figure 1:**
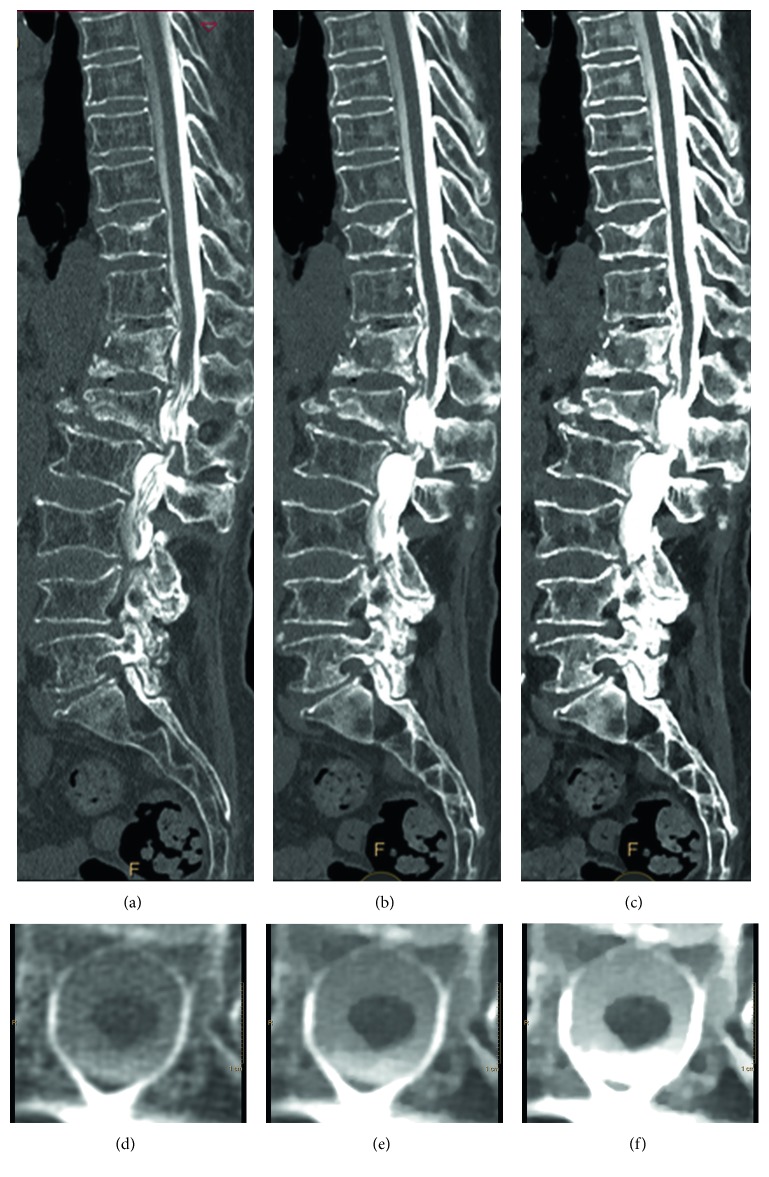
Computed tomography (CT) myelography images from a 73-year-old man (body weight 56 kg) with severe lumbar canal stenosis at the level of L1-L2 and multiple compression fractures (T10, T12, and L1). (a) Sagittal conventional CT myelography image. (b) Sagittal virtual monochromatic image (55 keV). (c) Sagittal virtual monochromatic image (40 keV). (d) Axial conventional CT myelography image at T6. (e) Axial virtual monochromatic image (55 keV). (f) Axial virtual monochromatic image (40 keV). The window setting was as follows: window width, 1250 Hounsfield units; window level, 250 Hounsfield units.

The CT myelogram also showed compression fractures of L2 and L3 with associated lumbar canal stenosis (Figure 1). Furthermore, we performed retrospectively spectral analysis using the workstation (Spectral Diagnostic Suite; Philips Healthcare, Best, Netherlands). The contrast attenuation in the spinal canal at 40 and 55 keV is better compared with that of the conventional images (Figure 1).

Additionally, we performed quantitative image analysis on the conventional CT image and spectral image data. We measured the mean attenuation of the spinal canal CT attenuation at the level of T6 using a circular region of interest (ROI_canal_). This ROI_canal_ was expected not to be so large that it included epidural fat or bone and spine. In addition, we also measured the CT attenuation of the spinal cord using a circular region of interest (ROI_spinal_) at the same level. Similarly, the ROI_spinal_ was expected not to be so large that it included the spinal canal. The reason why we selected the level of T6 was that the contrast of the spinal canal might be the lowest in the conventional images. In addition, we defined the standard deviation of attenuation at the iliopsoas muscle as the imaging noise. We measured the imaging noise at three sequential slices and averaged the results to minimize bias from single measurements. We also measured the contrast and the contrast-to-noise ratio (CNR) between the spinal cord and spinal canal. We defined the contrast as follows: ROI_canal_ − ROI_spinal_. The CNR was calculated as follows: (ROI_canal_ − ROI_spinal_)/image noise. We use a copy-and-paste function at the workstation to keep all measurements constant among the three kinds of images.

The results are shown in Table 1. The ROI_canal_ and CNR of virtual monochromatic images at 40 and 55 keV were significantly higher than those of the conventional CT images. In addition, the image noise of virtual monochromatic images at 40 and 55 keV was significantly lower than that of the conventional CT images. The conventional and virtual monochromatic images are shown in Figure 1.

We made a diagnosis of lumber stenosis at L1-L2, associated with clinical symptoms, and surgical treatment was proposed. The patient underwent laminectomy of L1-L2. He was discharged with symptomatic improvement after the operation.

## 3. Discussion

CT myelography is used to evaluate the spinal canal, spinal cord, spinal nerve roots, vertebrae, and discs when MRI is contraindicated. The amount of the contrast agent collecting in the upper thoracic and cervical thecal sacs may be less than that in the lumbar region. Therefore, conventional CT myelography can be suboptimal for the upper thoracic canal and cervical canal. We suggest the clinical usefulness of spectral analysis using the dual-layer spectral detector CT in improving the image quality in the upper thoracic and cervical canals. Previous report suggested that lower energy level (approaching the K-edge of iodine) increases the attenuation of iodine because of the predominance of the photoelectric effect [10]. Therefore, the iodine-containing spinal canal becomes hyperattenuated at lower energy levels on virtual monochromatic imaging [10]. Hence, in CT myelography, we might evaluate the upper thoracic canal and cervical canal more accurately using virtual monochromatic imaging compared with conventional CT.

In this case, the contrast between the spinal cord and spinal cavity in the thoracic canal was significantly greater on virtual monochromatic 40 keV and 55 keV images than that on conventional 120 kVp images (548.4 and 284.1 HU versus 192.0 HU). In addition, there was no significant difference in the image noise between virtual monochromatic 40 keV and 55 keV images and conventional 120 kVp images (28.8 and 23.9 HU versus 32.4 HU). The CNR of virtual monochromatic 40 keV and 55 keV images was significantly higher compared with that of conventional 120 kVp images (19.0 and 11.9 versus 4.9). Previous reports have suggested the usefulness of dual-layer spectral detector CT in reduction of beam-hardening artifact and improving the image quality of the coronary artery and abdominal CT scans [11–13]. However, to our knowledge, there have been no previous reports about the usefulness of dual-layer spectral detector CT in increasing the attenuation of the spinal canal in CT myelography.

There are two dual-energy CT systems in clinical use. The first method uses two orthogonal X-ray tubes set at different kVp levels with two separate detectors. The second method uses rapid kVp switching from a single X-ray source and a detector composed of two scintillation layers (the fast kVp switching method). The image of conventional dual energy CT technique cannot evaluate retrospectively adjusting energy level; therefore, it might be difficult in clinical use. However, the introduction of dual-layer spectral detector CT enables us to generate prospective and retrospective spectral images in all scans. Therefore, in case the contrast agent dose in the spinal canal during CT myelography is too low, retrospective spectral data analysis can increase the attenuation of the contrast agent in the spinal canal and might increase the diagnostic performance of the study.

The introduction of dual-layer spectral detector CT for CT myelography yields some clinical utility. First, lower energy levels on virtual monochromatic imaging can increase the CT attenuation of iodine in the spinal canal and might reduce the required contrast agent dose and concentration for CT myelography. The CT attenuation of the canal of 40 kVp image was 2.7 times higher compared with that of the conventional CT image (630.8 HU versus 230.4 HU). We supposed that CT attenuation of spinal canal is directly proportional to contrast agent dose. The contrast agent dose of dual-layer CT might be represented by the following formula: contrast agent dose (dual-layer CT) = contrast agent dose (conventional CT) × (230.4/630.8). Therefore, we might be able to reduce about 60% of the contrast agent while preserving the CT attenuation of the canal using the dual-layer spectral computed tomography compared with the compared conventional CT. Moreover, the reduction of contrast agent concentration might enable us to use a smaller gauge needle to inject the contrast agent. The smaller gauge needle might decrease technical complications such as dural tear, nerve root damage, CSF leak, and hemorrhage.

## 4. Conclusion

In conclusion, this case study suggested that dual-layer spectral detector CT increased the attenuation of the spinal canal in CT myelography and improved the image quality compared with conventional CT.

## Figures and Tables

**Table 1:** Quantitative image analysis.

	ROI_canal_	ROI_spinal_	Contrast	Image noise	CNR
Conventional CT image	230.4	38.4	192.0	32.4	4.9
Virtual monochromatic image (55 keV)	341.3	57.2	284.1	23.9	11.9
Virtual monochromatic image (40 keV)	638.3	89.9	548.4	28.8	19.0

## References

[1] Bischoff R. J., Rodriguez R. P., Gupta K., Righi A., Dalton J. E., Whitecloud T. S. (1993). A comparison of computed tomography-myelography, magnetic resonance imaging, and myelography in the diagnosis of herniated nucleus pulposus and spinal stenosis. *Journal of Spinal Disorders*.

[2] Spivak J. M. (1998). Degenerative lumbar spinal stenosis. *Journal of Bone and Joint Surgery*.

[3] Levine G. N., Gomes A. S., Arai A. E. (2007). Safety of magnetic resonance imaging in patients with cardiovascular devices: an American Heart Association scientific statement from the Committee on Diagnostic and Interventional Cardiac Catheterization, Council on Clinical Cardiology, and the Council on Cardiovascular Radiology and Intervention: endorsed by the American College of Cardiology Foundation, the North American Society for Cardiac Imaging, and the Society for Cardiovascular Magnetic Resonance. *Circulation*.

[4] Kaup M., Wichmann J. L., Scholtz J. E. (2016). Dual-energy CT-based display of bone marrow edema in osteoporotic vertebral compression fractures: impact on diagnostic accuracy of radiologists with varying levels of experience in correlation to MR imaging. *Radiology*.

[5] Srinivasan A., Hoeffner E., Ibrahim M., Shah G. V., LaMarca F., Mukherji S. K. (2013). Utility of dual-energy CT virtual keV monochromatic series for the assessment of spinal transpedicular hardware-bone interface. *American Journal of Roentgenology*.

[6] Kellock T. T., Nicolaou S., Kim S. S. Y. (2017). Detection of bone marrow edema in nondisplaced hip fractures: utility of a virtual noncalcium dual-energy CT application. *Radiology*.

[7] Sprung C., Fabian A. (1994). Pitfalls in computed tomography of the cervical and lumbar spine. *Neurosurgical Review*.

[8] Sakane M., Kim T., Hori M. (2014). Effects of high-concentration contrast material and low-voltage CT on contrast for multiphasic CT of the upper abdomen: comparison using the simulation with virtual monochromatic imaging obtained by fast-switch kVp dual-energy CT. *SpringerPlus*.

[9] Grams A. E., Sender J., Moritz R. (2014). Dual energy CT myelography after lumbar osteosynthesis. *RoFo: Fortschritte auf dem Gebiete der Rontgenstrahlen und der Nuklearmedizin*.

[10] Riederer S. J., Mistretta C. A. (1977). Selective iodine imaging using K-edge energies in computerized x-ray tomography. *Medical Physics*.

[11] Wellenberg R. H. H., Boomsma M. F., van Osch J. A. C. (2017). Quantifying metal artefact reduction using virtual monochromatic dual-layer detector spectral CT imaging in unilateral and bilateral total hip prostheses. *European Journal of Radiology*.

[12] Hickethier T., Baessler B., Kroeger J. R. (2017). Monoenergetic reconstructions for imaging of coronary artery stents using spectral detector CT: in-vitro experience and comparison to conventional images. *Journal of Cardiovascular Computed Tomography*.

[13] Oda S., Nakaura T., Utsunomiya D. (2017). Clinical potential of retrospective on-demand spectral analysis using dual-layer spectral detector-computed tomography in ischemia complicating small-bowel obstruction. *Emergency Radiology*.

